# Supplementary material for: Inverse Regulation of Lipocalin-2/24p3 Receptor/SLC22A17 and Lipocalin-2 Expression by Tonicity, NFAT5/TonEBP and Arginine Vasopressin in Mouse Cortical Collecting Duct Cells mCCD(cl.1): Implications for Osmotolerance
Source: Int J Mol Sci. 2019 Oct 30;20(21):5398. doi: 10.3390/ijms20215398 (PMC6862280; doi:10.3390/ijms20215398)
Supplement: Supplementary file 1 [file ijms-20-05398-s001.pdf]

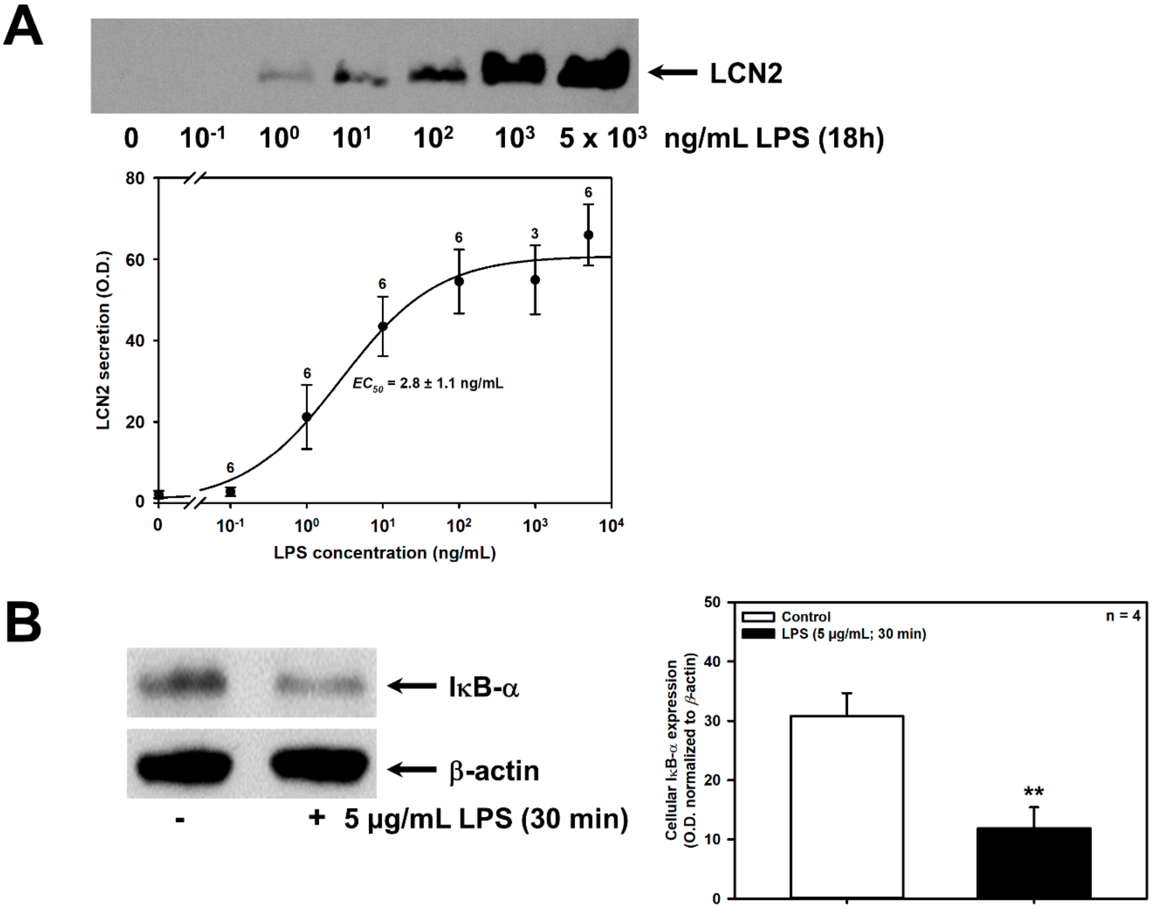

Supplementary Figure 1. A saturating concentration of LPS induces LCN2 secretion by activation of NF-κB dependent TLR4 signaling in cultured mCCD(cl.1) cells. (**A**) Cells were cultured for 24 h in normosmotic medium with FBSprior to treatment with different concentrations of LPS for 18 h in FBS-free medium. Medium was collected and LCN2 secretion determined by immunoblotting, as described in the Methods. The dose-response curve of the effect of different LPS concentrations on LCN2 secretion was fitted with the Sigma Plot 12.5 spreadsheet program assuming a sigmoidal function and using the Hill equation. Data show means ± SEM of 3-6 experiments. (**B**) mCCD(cl.1) cells were cultured as described above and treated with ± LPS (5 µg/ml) for 30 min in medium without FBS prior to washing, scraping and homogenization by sonication in isosmotic sucrose buffer supplemented with protease inhibitors for immunoblotting. Cellular IκB-α protein was normalized to β-actin. Data show means ± SEM of 4 experiments. Statistical analysis determines the effect of LPS on cellular IκB-α protein using unpaired *t*-test. For a definition of asterisks, see "statistics" in the Methods.
